# NCBoost v2: a classifier for non-coding single-nucleotide variants in Mendelian diseases

**DOI:** 10.1093/bioinformatics/btag138

**Published:** 2026-03-25

**Authors:** Barthélémy Caron, Antonio Rausell

**Affiliations:** Clinical Bioinformatics Laboratory, Université Paris Cité, INSERM UMR1163, Imagine Institute, Paris F-75006, France; Clinical Bioinformatics Laboratory, Université Paris Cité, INSERM UMR1163, Imagine Institute, Paris F-75006, France; Service de Médecine Génomique des Maladies Rares, Fédération de Génétique et Médecine Génomique, AP-HP, Necker Hospital for Sick Children, Paris F-75015, France

## Abstract

**Motivation:**

The current diagnostic rate of rare diseases through whole-genome sequencing has stabilized at around 30% on average, highlighting the need for improved computational scores to identify pathogenic variants. In 2019, we developed NCBoost, a supervised-learning approach that mined a comprehensive set of sequence constraint features and proved particularly well suited to identifying high-effect pathogenic non-coding variants in genetic diseases. Since its first release, the substantial increase in the number of variants available for training, as well as the enhanced capacity to detect purifying selection signals from large-scale genome sequencing projects, motivated an update of NCBoost.

**Results:**

We implemented NCBoost v2, a pathogenicity score for non-coding single-nucleotide variants, trained on the largest set of curated pathogenic variants in monogenic Mendelian diseases available to date. It leverages conservation features computed from recent large-scale genomic consortia such as Zoonomia and gnomAD, and incorporates recent splice-altering predictive scores. NCBoost v2 outperformed alternative state-of-the-art methods in a variety of scenarii, providing more consistent scores across non-coding genomic regions and fine-tuning the scoring of pathogenic splice-altering variants in Mendelian disease genes.

**Availability and implementation:**

NCBoost v2 software is implemented in Python 3.10 and is freely available under the GNU General Public License Version 3 at https://doi.org/10.5281/zenodo.16029049 and https://github.com/RausellLab/NCBoost-2, together with precomputed scores for the human genome assembly GRCh38.

## 1 Introduction

Rare diseases affect approximately 5%–8% of the global population (Kernohan and Boycott 2024), with more than 7000 distinct disorders described to date (OMIM.ORG, https://www.omim.org/, last accessed on 2 July 2025), about 80% of which are estimated to have a genetic basis. Whole genome sequencing has become a first-line clinical diagnosis test. Its adoption in national genomic medicine plans has demonstrated its added value in improving diagnostic yield, including the identification of novel causal variants in non-coding genomic regions, among other factors (PFMG2025 contributors 2025). Yet, whole genome sequencing (WGS) yields a molecular diagnosis in only 30%–50% of suspected Mendelian cases, depending on disease type (Kaur et al. 2024). The important fraction of unresolved patients highlights the need for improved methods to prioritize genetic variants, particularly in non-coding regions, which make up 98% of the human genome. A number of computational scoring methods have contributed to the identification of causal non-coding variants (Fu et al. 2014, Kircher et al. 2014, Zhou and Troyanskaya 2015, Ionita-Laza et al. 2016, Smedley et al. 2016), including our NCBoost method (Caron et al. 2019). NCBoost is a supervised learning approach trained to discriminate pathogenic from benign non-coding single-nucleotide variants (SNVs) by leveraging a comprehensive set of interspecies conservation features, as well as recent and ongoing sequence constraint signals in the human lineage. NCBoost proved to be particularly well-suited for the assessment of monogenic genetic diseases, which are often driven by high-effect causal variants subject to purifying selection. NCBoost was referred to in the recommendations for clinical interpretation of variants found in non-coding regions of the genome published in Ellingford et al. (2022). Since its first release in 2019, the widespread adoption of WGS has translated in a significant increase in the number of reported causal variants in non-coding regions in reference databases for rare disease patients such as ClinVar (Landrum et al. 2014) and HGMD professional (Stenson et al. 2020). In addition, recent large-scale sequencing projects in vertebrates, mammals, primates as well as of the general population, are permitting the identification of purifying selection signals in genomic sequences with enhanced resolution (Zoonomia Consortium et al. 2020, Chen et al. 2024). Furthermore, novel computational methods have greatly improved the identification of splice-altering variants (Jaganathan et al. 2019, Cheng et al. 2019, Wagner et al. 2023) and have been integrated within genetic diagnosis protocols (Walker et al. 2023). To take advantage of the previous developments, we present NCBoost v2, an updated version of the NCBoost approach on the newer human genome assembly hg38 that is trained on a significantly larger set of high-confidence pathogenic non-coding SNVs associated with monogenic Mendelian diseases (MMDs), annotated with more comprehensive conservation features computed on recent large-scale genomic projects as well as with splice-altering scores, and thoroughly tested and validated in multiple scenarii.

## 2 Methods

NCBoost v2 is a supervised learning algorithm using extreme gradient boosting (XGBoost) and trained to discriminate non-coding pathogenic SNVs associated with MMDs from non-pathogenic SNVs. Non-coding pathogenic variants were obtained from HGMD professional (Stenson et al. 2020) and ClinVar (Landrum et al. 2014). Extensive curation steps, including filtering variants with conflicting annotations, complex or somatic disease architectures, or evidence of homozygosity in population datasets, were followed to obtain a high-confidence set of *N* = 2336 non-coding SNVs causing MMDs, collectively involving 765 unique genes (Figure 1 and Supplementary Text, available as supplementary data at *Bioinformatics* online). This dataset is significantly larger than the analogous set available at the time of the first release of NCBoost (737 variants associated with 283 unique genes). Random common variants were sampled from dbSNP (Sherry 2001) and used as the negative training set.

To avoid cross-contamination between training and downstream application sets, NCBoost v2 was trained as a bundle of 10 independent models as follows: protein-coding genes were randomly split into 10 genomic partitions, equally stratified across chromosomes, with each containing a similar number of genes with and without high-quality non-coding pathogenic variants. Each positive non-coding SNV was paired with 10 random negative SNVs from the same genomic partition and the same type of genetic region (upstream, 5′UTR, intronic, 3′UTR, downstream, and intergenic). Throughout these steps, a maximum of 1 positive and 1 negative variant per gene were sampled, while maintaining a global 1:10 positive-to-negative ratio in the training set (Supplementary Text, available as supplementary data at* Bioinformatics* online).

Variants were annotated with 58 features, including 26 new ones compared to the initial NCBoost version, belonging to four different feature categories (Table 1 and Supplementary Text, available as supplementary data at *Bioinformatics* online): (i) long-term interspecies sequence conservation features, including recent large-scale alignment of 241 vertebrate genomes and 43 primate genomes from the Zoonomia project (Zoonomia Consortium et al. 2020); (ii) recent and ongoing human sequence constraint features, computed on more than 76 000 genomes from GnomAD v4.1, representing nine major subpopulations (Chen et al. 2024); (iii) conservation features of the associated protein-coding gene, including both interspecies and in-human sequence conservation, such as the *loeuf* score, computed on more than 730 000 individuals from GnomAD (Karczewski et al. 2020); and (D) sequence context features, such as GC and CpG content around the position, the type of overlapping non-coding region and the maximum SpliceAI score at the variant and position levels, in order to inform the classifier about its splice-altering potential (Supplementary Text and Figure 2, available as supplementary data at *Bioinformatics* online).

**Table 1:** The table reports the ability of NCBoost v2 to discriminate between pathogenic and non-pathogenic non-coding single-nucleotide variants, together with two alternative state-of-the-art methods: ReMM and CADD.^a^

	Region	N patho-genic	N benign	NCBoost v2		ReMM		CADD	
				AUROC	AUPR	AUROC	AUPR	AUROC	AUPR
**Genomic-partition cross-validation**	**Global**	**765**	**7650**	**0.94**	**0.79**	**0.71**	**0.25**	**0.78**	**0.49**
	Upstream	61	610	0.87	0.52	0.84	0.49	0.84	0.43
	UTR5	133	1330	0.85	0.45	0.72	0.25	0.72	0.26
	Intronic	475	4750	0.98	0.91	0.72	0.27	0.80	0.61
	UTR3	77	770	0.83	0.50	0.69	0.24	0.73	0.34
	Downstream	9	90	0.91	0.63	0.83	0.45	0.84	0.35
	Intergenic	10	100	0.82	0.32	0.76	0.40	0.74	0.47
**Testing**	**Global**	**687**	**6870**	**0.88**	**0.54**	**0.79**	**0.43**	**0.78**	**0.44**
	Upstream	177	1770	0.90	0.65	0.86	0.57	0.86	0.58
	UTR5	252	2520	0.81	0.34	0.77	0.43	0.76	0.42
	Intronic	156	1560	0.97	0.79	0.80	0.45	0.76	0.46
	UTR3	80	800	0.84	0.51	0.78	0.32	0.77	0.44
	Downstream	9	90	0.99	0.91	0.82	0.67	0.90	0.74
	Intergenic	13	130	0.86	0.64	0.84	0.65	0.81	0.60
**Validation**	**Global**	**158**	**1580**	**0.96**	**0.84**	**0.71**	**0.25**	**0.82**	**0.54**
	Upstream	1	10	1.00	1.00	1.00	1.00	1.00	1.00
	UTR5	18	180	0.90	0.50	0.78	0.32	0.73	0.45
	Intronic	123	1230	0.97	0.91	0.72	0.28	0.85	0.60
	UTR3	18	180	0.81	0.39	0.69	0.29	0.67	0.25
	Downstream	1	10	1.00	1.00	0.40	0.07	0.60	0.10
	Intergenic	3	30	0.87	0.72	0.61	0.41	0.73	0.56

a The area under the receiver operating characteristic (AUROC) curve and the area under the precision-recall (AUPR) curve are reported for each method across three evaluation settings: genomic partition cross-validation, independent testing, and validation (Methods and Supplementary Text, available as supplementary data at *Bioinformatics* online). For each setting, the global AUROC and AUPR values are reported, along with those corresponding to the different region types evaluated independently. The total number of pathogenic and non-pathogenic variants is reported for each setting (bold), as well as broken down by region type.

## 3 Results

NCBoost v2 was first cross-validated on each of the 10 genomic partitions, iteratively left out during the training phase, collectively adding a total of 765 high-quality non-coding pathogenic variants and the corresponding region-matched set of 7650 random common non-coding SNVs (Table 1, Figure 3 and Supplementary Text, available as supplementary data at *Bioinformatics* online). NCBoost v2 achieved an area under the receiver operating characteristic (AUROC) curve of 0.94 and a precision-recall (AUPR) curve of 0.79, outperforming NCBoost v1 as well as two alternative methods, representative of the state-of-the-art, such as ReMM (Schubach et al. 2022) and CADD (Schubach et al. 2024, Figure 3, available as supplementary data at *Bioinformatics* online, available as supplementary data at *Bioinformatics* online). Consistent results were obtained when evaluating on random sets of rare non-coding SNVs as the negative set (Figure 4, available as supplementary data at *Bioinformatics* online). NCBoost v2’s superiority was consistent across all genomic region types, independently considered (Table 1). Outperformance of NCBoost v2 was also consistently observed when excluding SpliceAI from the feature set, whether considered globally or for each genomic region (Table 2, available as supplementary data at *Bioinformatics* online). For the remainder of this work, NCBoost v2 refers to the framework trained with SpliceAI included as a feature.

Consistent with our previous observations (Caron et al. 2019), per-gene region biases were observed across common SNVs without clinical assertions, with the 5′ UTR showing the strongest shift towards higher values, suggesting that the regulatory region where a variant maps systematically biases the scores (Figure 5 and Supplementary Text, available as supplementary data at *Bioinformatics* online). However, only in the case of NCBoost v2 were the distributions of median scores per gene for common SNVs in 5′ UTRs significantly different (with less severe scores) from those of pathogenic SNVs in the 5′ UTR as well as in upstream, intronic, 3′ UTR, and downstream regions of the associated genes, while ReMM and CADD failed to overcome such bias in several of those regions or did so with weaker *P*-values (Table 3, available as supplementary data at *Bioinformatic*s online).

We further evaluated NCBoost v2 on an independent test set of *N* = 687 high-confidence, manually curated non-coding pathogenic variants reported at the time of NCBoost’s first release (Caron et al. 2019), as well as in a validation set of *N* = 158 non-coding pathogenic variants reported after January 2025 (Supplementary Text, available as supplementary data at *Bioinformatics* online). NCBoost’s capacity to discriminate pathogenic from non-pathogenic variants generalized both in the test and validation sets (Figures 6 and 7, available as supplementary data at *Bioinformatics* online), was consistent across all gene regions evaluated, and systematically outperformed alternative scores (Table 1).

To test NCBoost v2 performance in more realistic clinical diagnosis scenarii, we simulated disease genomes by introducing within each genome of 100 healthy individuals, independently, each of the non-coding pathogenic variants from the validation set along with a random common variant mapping to the same non-coding region of the same gene (Supplementary Text, available as supplementary data at *Bioinformatics* online). While the three evaluated scores ranked the non-coding pathogenic variants significantly higher than the matched common variants (one-sided paired Wilcoxon test: NCBoost v2 *P*-value = 1.21e−23, ReMM *P*-value = 2.52e−08, and CADD *P*-value = 55.82e−17), NCBoost v2 showed the best within-individual ranking of non-coding pathogenic variants (median = 99.93%), significantly outperforming ReMM (median = 92.95%, one-sided paired Wilcoxon test *P*-value = 7.66e−18) and CADD (median = 98.32%, *P*-value = 1.37e−15, Figure 8, available as supplementary data at *Bioinformatics* online).

Finally, given the high importance of SpliceAI as a predictive feature in the NCBoost v2 model (Figure 2, available as supplementary data at *Bioinformatic*s online), we evaluated whether NCBoost v2 provides an added value compared to using SpliceAI as a raw score. To do this, for each protein-coding gene, we obtained the top-scoring SpliceAI non-coding position’s score along with its corresponding NCBoost v2 score. We then compared the gene rank percentiles of such scores across *N* = 6128 MMD genes and *N* = 12 640 non-disease genes. While the distribution of both scores were significantly higher across MMD genes than non-disease genes (two-sided Mann–Whitney–Wilcoxon rank-sum test *P*-value = 1.59e−22 and 1.96e−114, for SpliceAI and NCBoost v2, respectively), NCBoost v2 provided significantly higher ranks (i.e. more pathogenic) to MMDGs splice-altering variants than SpliceAI (two-sided Mann–Whitney–Wilcoxon rank-sum test *P*-value = 3.44e−21, and two-sided Wilcoxon paired signed-rank test *P*-value = 4.09e−22, Figure 9 and Supplementary Text, available as supplementary data at *Bioinformatics* online).

## 4 Discussion

NCBoost v2 proved to be a valuable supervised-learning approach for identifying pathogenic non-coding SNVs in MMDs across diverse independent test and validation settings. NCBoost outperformed reference state-of-the-art scores recently updated for the newer human genome assembly, hg38. Its superiority was consistent across all genomic region types, considered independently. Notwithstanding, consistent with our previous observations (Caron et al. 2019), per-gene region biases were still observed for all evaluated scores. Inspection of feature distributions across regions revealed heterogeneous trends (Figure 10, available as supplementary data at *Bioinformatics* online), suggesting that per-gene regional score biases and potential mitigation strategies remain open questions requiring further investigation. However, NCBoost v2 was the only score capable of assigning more severe scores to pathogenic than to non-pathogenic variants, independently of the gene region considered (i.e. upstream, 5′UTR, intronic, 3′UTR, and downstream regions). Finally, the incorporation of SpliceAI scores as features into the NCBoost v2 framework resulted in a score that better discriminates splice-altering variants in MMD genes compared to non-disease genes, improving the raw SpliceAI values. These results suggest that NCBoost v2’s integration of variant- and gene-level conservation features helps fine-tuning SpliceAI predictions for improved application in rare disease genomes.

Rather than as a stand-alone decision criterion, the NCBoost v2 score is intended to be used within comprehensive genetic diagnostic pipelines further evaluating the consistency between altered genomic elements and observed clinical phenotypes. Its interpretation should thus follow existing expert recommendations for the clinical assessment of non-coding variants, which extensively discuss the consideration of bioinformatic prediction scores as well as ACMG/AMP-based variant classification frameworks in genetic diagnostics (Ellingford et al. 2022, Walker et al. 2023).

## Supplementary Material

btag138_Supplementary_Data

## Data Availability

NCBoost v2 software is freely available at https://github.com/RausellLab/NCBoost-2, together with partial training datasets allowing training and testing of the model and precomputed scores for the human genome assembly GRCh38.
