# Supplementary figures and images for: NCBoost v2: a classifier for non-coding single-nucleotide variants in Mendelian diseases
Source: Bioinformatics. 2026 Mar 25;42(5):btag138. doi: 10.1093/bioinformatics/btag138 (PMC13141147; doi:10.1093/bioinformatics/btag138)

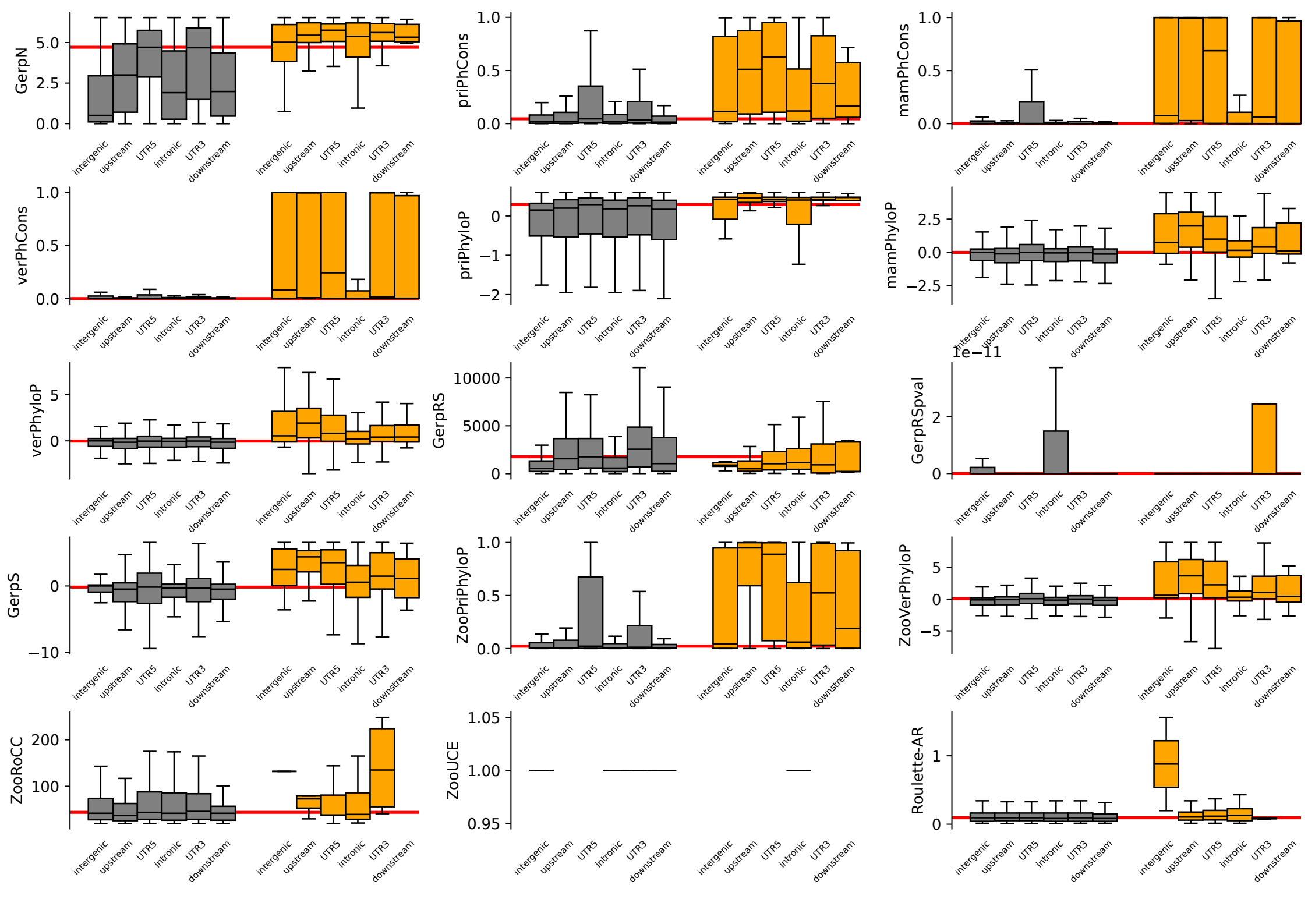

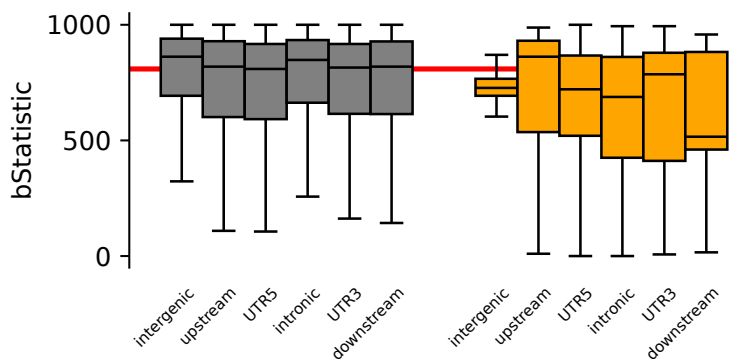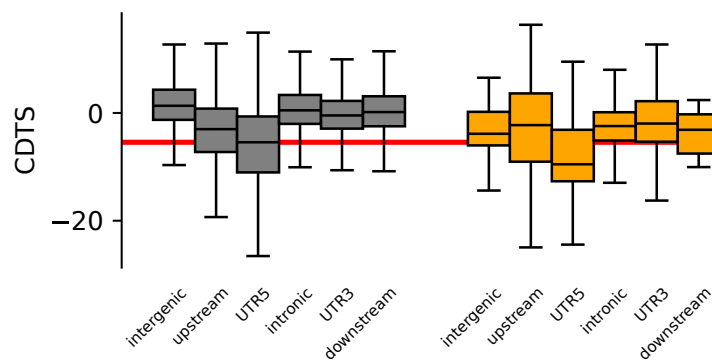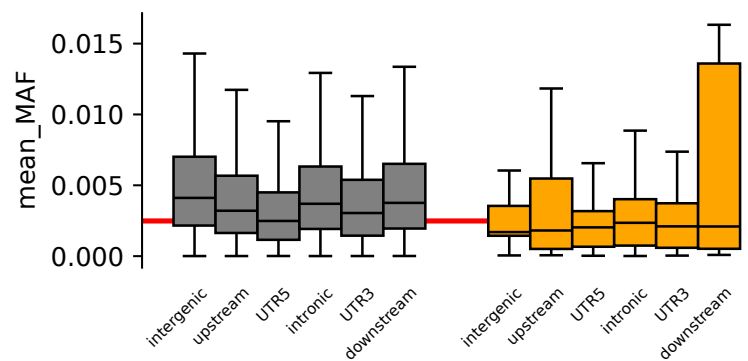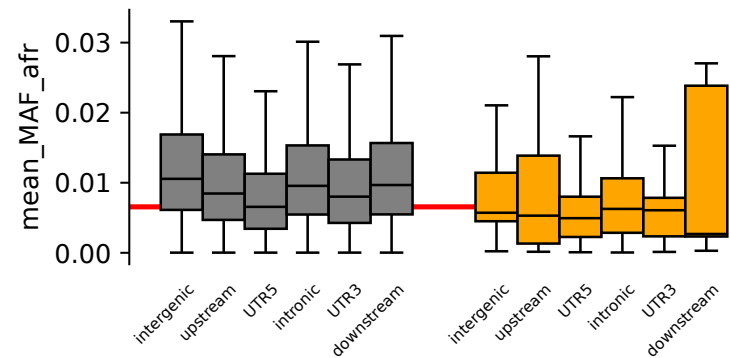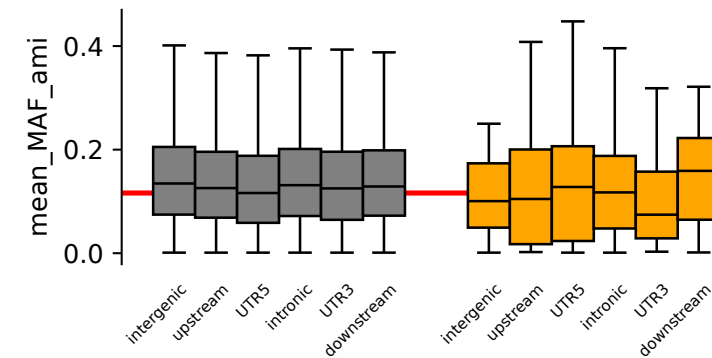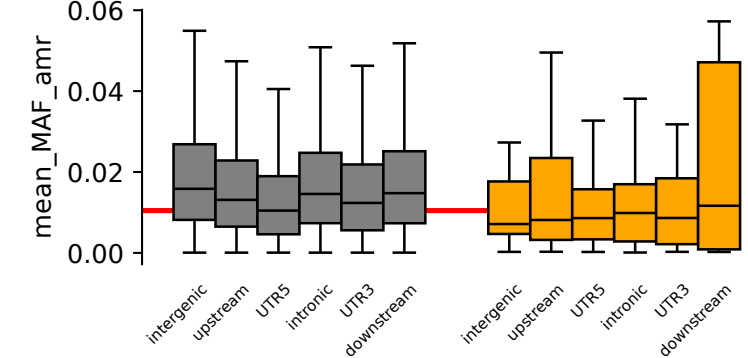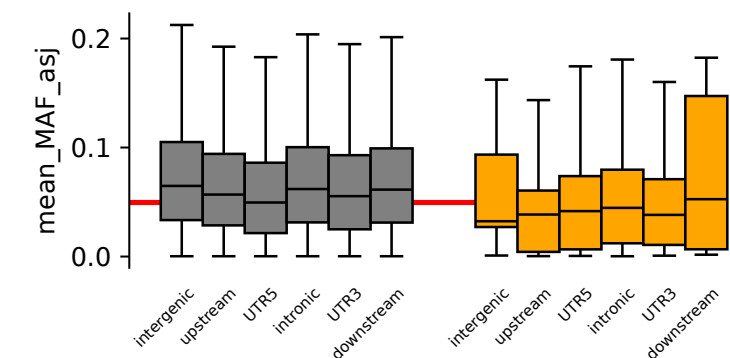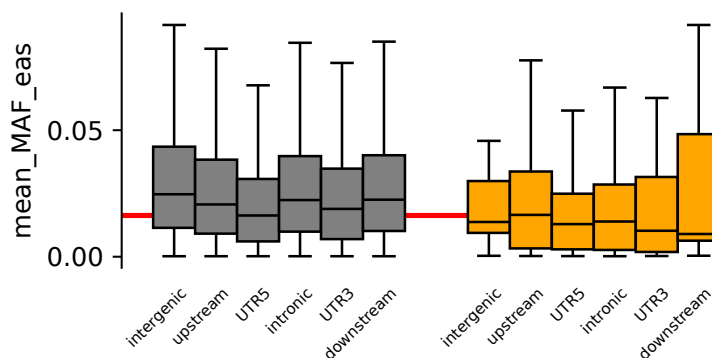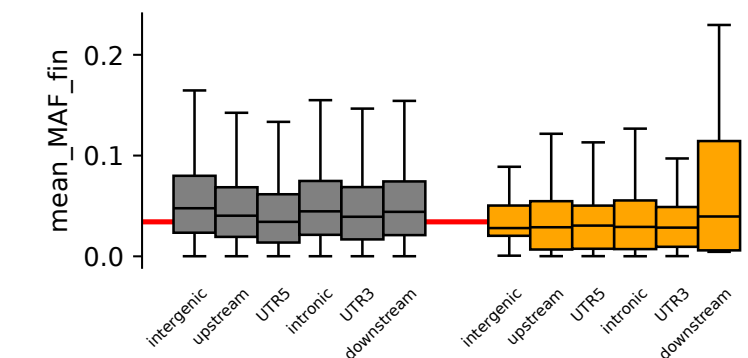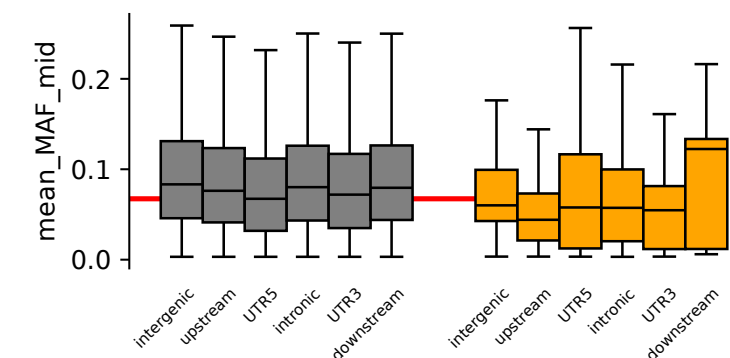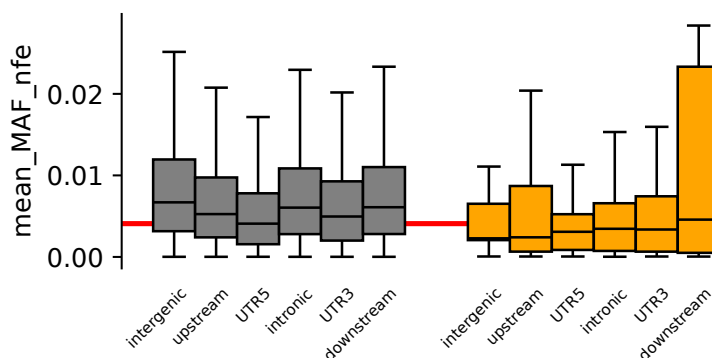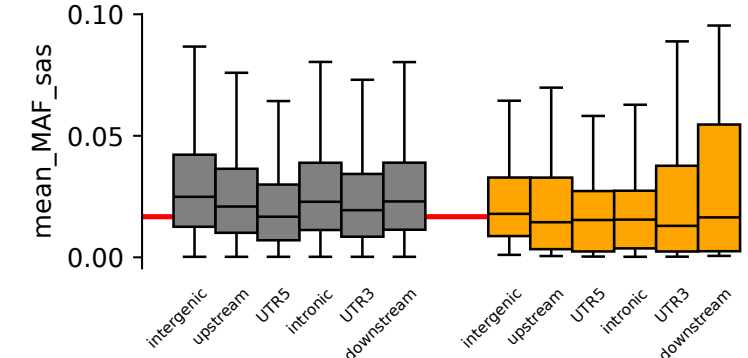

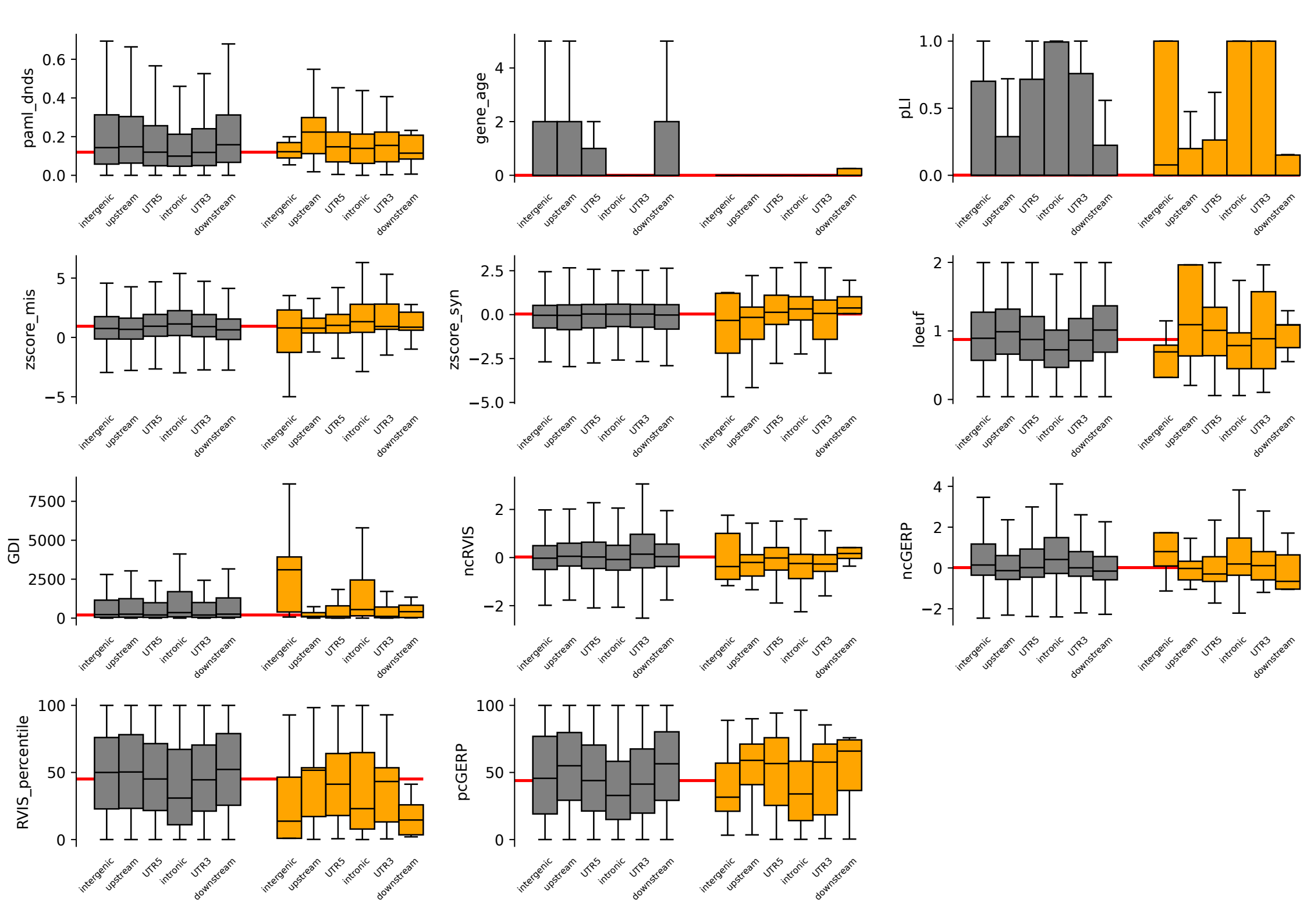

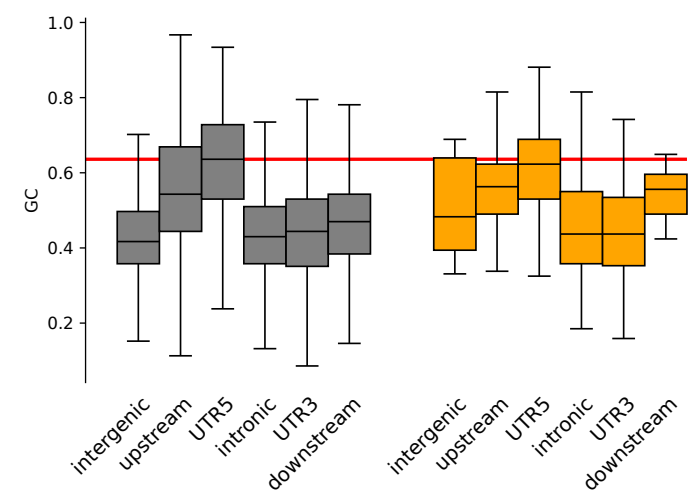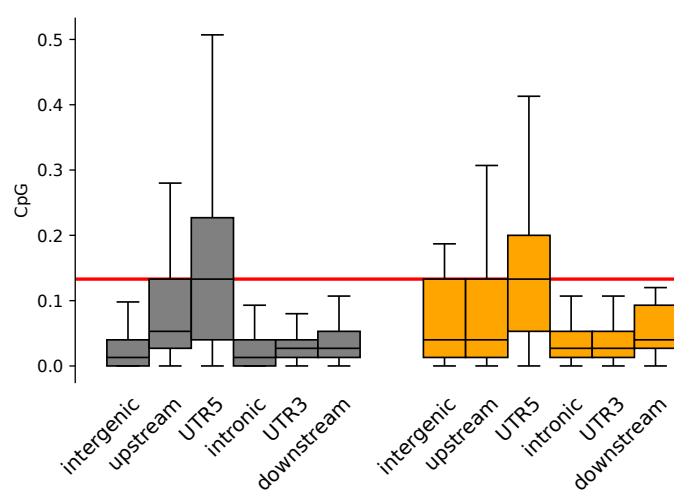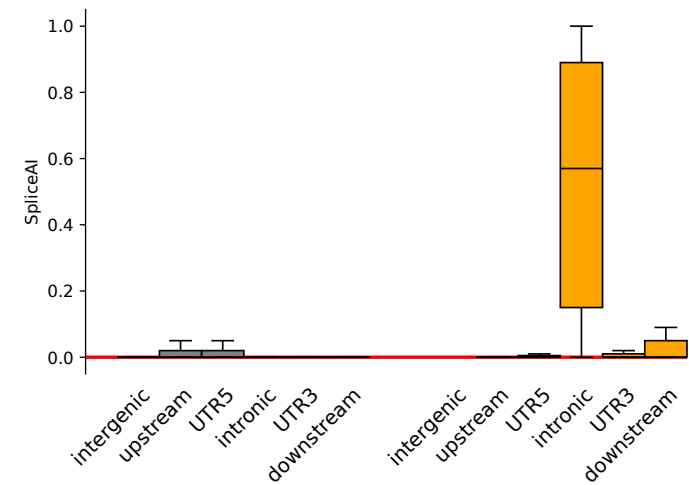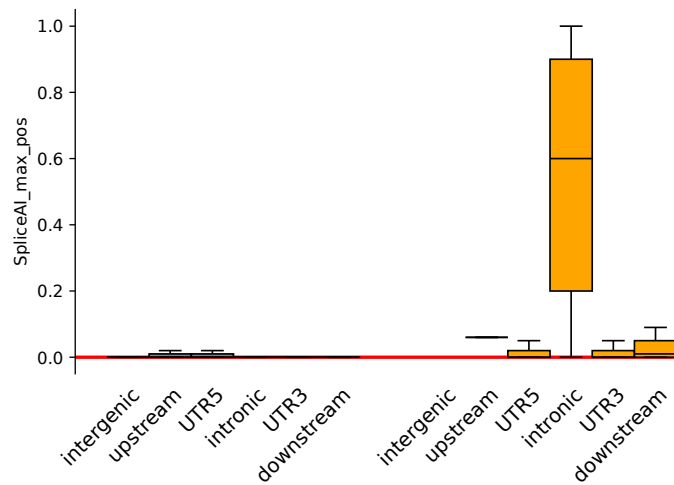

Supplement: btag138_Supplementary_Data [file btag138_supplementary_data.zip › FigureS10.pdf]
